# On the efficacy of facial masks to suppress the spreading of pathogen-carrying saliva particles during human respiratory events: Insights gained via high-fidelity numerical modeling

**DOI:** 10.18103/mra.v12i5.5441

**Published:** 2024-05-27

**Authors:** Hossein Seyedzadeh, Jonathan Craig, Ali Khosronejad

**Affiliations:** a Department of Civil Engineering, Stony Brook University, Stony Brook, NY 11794, USA

**Keywords:** Bio-fluids dynamics, Vortex dynamics, Saliva particle transport, Human breathing, Coughing

## Abstract

Respiratory fluid dynamics is integral to comprehending the transmission of infectious diseases and the effectiveness of interventions such as face masks and social distancing. In this research, we present our recent studies that investigate respiratory particle transport via high-fidelity large eddy simulation coupled with the Lagrangian particle tracking method. Based on our numerical simulation results for human respiratory events with and without face masks, we demonstrate that facial masks could significantly suppress particle spreading. The studied respiratory events include coughing and normal breathing through mouth and nose. Using the Lagrangian particle tracking simulation results, we elucidated the transport pathways of saliva particles during inhalation and exhalation of breathing cycles, contributing to our understanding of respiratory physiology and potential disease transmission routes. Our findings underscore the importance of respiratory fluid dynamics research in informing public health strategies to reduce the spread of respiratory infections. Combining advanced mathematical modeling techniques with experimental data will help future research on airborne disease transmission dynamics and the effectiveness of preventive measures such as face masks.

## 1. Introduction

The effects of COVID-19 are ubiquitous. As of 11 February 2024, there have been 774,631,444 reported COVID-19 cases worldwide, 7,031,216 of which were fatal^1^. Whereas COVID-19 did not result in as many deaths as circulatory diseases and cancer, it substantially raised the number of deaths in Organization for Economic Co-operation and Development (OECD) countries by 1.5 million between 2019 and 2021^2,3^. This is almost 1.5 million more than in 2019, largely due to COVID-19. The virus’ global case fatality rate decreased from 20% in early 2020 to 0.3% by August 2022^4^; nevertheless, COVID-19 has remained a significant cause of death. In the United States, COVID-19 was the third most common cause of death in 2021, fourth in 2022, and tenth in 2023^5^. Before the onset of the COVID-19 pandemic, various publications purported the face masks’ efficacy in restricting expiratory particle transport and, thereby, virus transmission^6–8^. By meta-analysis of 21 studies, Liang et al.^7^ summarized that face masks effectively reduced the spread of respiratory viral infections, including COVID-19. In a rapid systematic review of observational studies, Boulos et al.^6^ likewise noted that mask-wearing and public mandates generally reduced viral transmission. Even so, both studies revealed a dearth of high-quality medical studies about the efficacy of mask-wearing in public policy and environments, and the results of the studies varied considerably in magnitude and precision.

The preliminary social distancing and mask-wearing guidelines of the World Health Organization (WHO) and the United States Center for Disease Control (CDC) reflected the disparity and dearth of concrete understanding about face masks’ protective role in virus transmission^9^. Specifically, Bourouiba^10^ clarified that the preliminary guidelines were based on outdated experimental research^11^ that did not consider critical fluid-flow physics such as particle size and turbulence, which could affect the efficacy of mask-wearing. Fontes et al.^12^ also illustrated that a more current study by Olsen et al.^13^ about SARS-CoV 2 transmission in an airplane lacked multiphase analysis of airflow and particle transport.

The knowledge gap in the role of particle transport within virus transmission catalyzed several fluid dynamics studies about COVID-19 in diverse scenarios of fluid flow: over general cases of respiration, encompassing breathing, talking, coughing, and sneezing; for various physiological and biological factors, such as health, anatomy, stress, sex, and age; and with multiple environmental factors, including turbulence, temperature, and humidity^12,14–29^. Generally, these studies employed computational fluid dynamics (CFD) for in silico experiment of fluid flow problems, which allows precise tracking of specific particle data such as size and location along with testing of complex problems without the need for physical experimental setup^30^. Even though high rates of particle generation and exhalation characterize coughing and sneezing, Mittal et al.^31^ acknowledged that talking and breathing mainly constitute respiratory particle transport in a daily context. Further, Dbouk and Drikakis^25^ demonstrated with Reynolds average Navier-Stokes (RANS) that coughed saliva droplets could quickly travel over several meters in a turbulent outdoor setting. Both studies of Dbouk and Drikakis^25,26^ underscored that saliva droplets exhibit an initial size distribution which affects the droplets’ flow physics. Generally, gravity is the dominant force for bigger respiratory droplets, whereas aerodynamic forces such as lift and drag are the dominant forces for smaller droplets^27^. Whereas bigger droplets are capable of containing higher quantities of virus, smaller droplets can be more potent for virus transmission due to their much longer suspension time in the air^12,16,32^. With an Eulerian-Lagrangian (EL) framework and detached eddy simulation (DES), Fontes et al.^12^ observed vorticity and recirculation of saliva particles in a sneeze, which affected suspension time and travel distance. In addition, Li et al.^29^ highlighted that evaporation could alter saliva particle transport and virus transmission with changing particle size, which temperature and humidity influence. The saliva particles’ physiochemical properties, including concentrations of solid, salt, and mucin, also influence their evaporation^19,27–29^.

Over the course of the pandemic, CFD simulations became more comprehensive and representative of realistic scenarios for respiratory particle transport: a bus, a Boeing 737 plane, a conference room, an office, a classroom, a restaurant, an escalator, a doorway, and a generic indoor setting^33–44^. Particular studies have focused on potential scenarios of virus transmission in medical settings such as a hospital isolation room^32,45^ or an operating room^46^. Most of these studies^33,35,37,40–42^ employed an EL approach for tracking of discrete particles instead of an Eulerian-Eulerian (EE) approach for the flow field; though, Islam et al.^47^ detected no difference in influence from either EL or EE on particles’ deposition patterns. Additionally, many studies illustrated the vital role protective measures such as screens, barriers, and ventilation can provide in cutting the spread of infected saliva particles^33,35,36,38,44,45,48^. Overall, the novel consideration of particle transport enabled both experimental and computational studies to corroborate face masks’ utility in curtailing virus transmission^16,24,26,43,44,49^.

Along with the growing understanding of particle transport physics in the context of COVID-19 virus transmission, CFD simulations advanced to consider more sophisticated features. Namely, Haller^50^ recognized the presence of Lagrangian coherent structures (LCS), material lines known as manifolds that delineate two-dimensional invariant boundaries where particles do not cross. Thence, several studies have observed that LCS manifest as flows throughout natural and engineered systems, including medical applications^51–59^. Oaks et al.^60^ innovated by applying LCS to describe the biological flow in normal human breathing, and Seyedzadeh et al.^61^ elaborated the initial study by identifying other intricate three-dimensional (3D) structures such as jet in crossflow (JICF)^62^ and counter-rotating vortex pairs (CRVP). Exploration of LCSs has guided similarly innovative studies in medicinal settings^32,46,63^. Amahjour et al.^32^ applied Lagrangian descriptors to classify particle transport evolution in a hospital isolation room with varied air conditioning and sanitizing device placement. With a finite-time Lyapunov exponent field, Kumar et al.^46^ exposed regions of vortex formation and accumulation to guide the optimization of removing surgical smoke during laparoscopy. Lagrangian descriptors enabled Abdallah et al.^63^ to extract LCSs from aortic regurgitation, highlighting complex flow structures in the interaction between mitral inflow and the regurgitant jet. Nonetheless, Badza et al.^64^ conceded that although methods for fully dimensional coherent flows are relatively robust, uncertainties within the Eulerian data ultimately decide whether a given LCS method is robust. Moreover, studies such as those of Lagares and Araya^56^ and Hayat et al.^65^ have indicated that there is yet much potential for improvement of LCS computation in parallel programming as well as explicit particle tracking, respectively.

Seyedzadeh et al.^61^ also combined nasal breathing into the original oral breathing model of^60^, which is a reflection of high-fidelity CFD studies’ continuous progression for a comprehensive depiction of biological flows. After all, particle deposition first occurs in the nasopharyngeal region^30^ whose geometrical structures cause different flow patterns in inspiration and expiration^66^; yet, it is important to note that previous studies incorporated nasal airflow to model particle deposition within the human body. Through RANS simulation of the Carleton-Civic model, Liu et al.^67^ measured that big particles of between 5.77 and 10.0 *μm* in size most significantly deposit along the first 30% of the nasal cavity’s length. Islam et al.^47^ determined that although droplet deposition density is higher for mouth breathing, nose breathing permits a broader droplet distribution throughout the respiratory system. Even though such studies^22,47,67^ examined the nose effect in internal respiration, preliminary fluid dynamics modeling of COVID-19 did not commonly incorporate the nose effect in external respiration^12^. As evidenced by the work of Fontes et al.^12^, the nasal effect can significantly raise droplet content in a droplet cloud. In a high-fidelity CFD simulation during high-velocity nasal insufflation, Leonard et al.^30^ measured that a surgical mask could capture 88.8% of exhaled particulate mass. Through a hybrid RANS-LES turbulence method and a discrete phase model, Salati et al.^20^ visualized nasal airflow in a sneeze as two parallel jets in a shared flow, which interfere with each other and expand. Ijaz et al.^48^ ran CFD simulations with a discrete phase model to optimize visor geometry in high-flow nasal oxygen therapy, in which they achieved 38–47% improvement of design efficiency. Behera et al.^68^ examined elevated JICF by direct numerical simulation (DNS) with phase-averaging, observing vortex systems that Seyedzadeh et al.^61^ detected in the interaction of the mouth jet and nose cross flow.

In this paper, we recapitulate our findings pertaining to high-fidelity CFD modeling of human respiratory events, including normal breathing and coughing, whereby we reflect the evolving understanding of particle transport from the perspective of fluid dynamics and LCS. We employ a high-resolution computational grid system to perform DNS of the air-saliva mixture during human respiratory events under both indoor (room) and outdoor conditions. The obtained airflow field is employed to solve for the individual saliva particles’ Lagrangian trajectories. The evaporation process of the water content of the particles is monitored by reducing the diameter of the particles as a function of time. Due to the difference in density between the saliva particle cloud and the air, the effect of density stratification on flow dynamics is considered using the Boussinesq approximation. Based on the in-silico experimentation of indoor human respiratory events, the simulations overall show that common facial masks can limit the traveling distance of saliva particles to about 0.5 m from the person, while unmasked breathing or coughing can lead to particle spreading to distances beyond 2 m in only a few minutes. In the presence of a mild breath, outdoor human respiratory events appear unaffected by mask-wearing, and saliva particles can traverse distances farther than 2 m in a matter of seconds.

The paper is organized according to the following description: Section 2 encompasses the material and methods, containing the governing equations and boundary conditions. Section 3 refers to the human anatomy and face mask geometry description. Section 4 includes the computational setup description of the present study. Section 5 contains the results and discussion of our studies. Section 6 presents the conclusions drawn from these results.

## 2. Material and Methods

In this section, we describe the numerical model employed for simulating the flow dynamics, encompassing the governing equations for Eulerian and Lagrangian flow dynamics, air-saliva mixture convection-diffusion, evaporation effect, and boundary conditions requisite for our simulations, with the aim of elucidating the underlying flow physics.

### 2.1. EQUATIONS OF FLUID MOTION

The Eulerian equations governing the motion of the air–saliva mixture comprise the filtered continuity and Navier–Stokes equations expressed in generalized curvilinear coordinates using the compact tensor notation: i,j=1,2,3; repeated indices imply summation. They can be represented as follows^69^:

(1) $$J\frac{\partial U^{j}}{\partial \xi^{j}}=$$

(2) $$\frac{1}{J}\frac{\partial U^{j}}{\partial t}=\frac{\xi_{l}^{i}}{J}\left\{-\frac{\partial \left(U^{j}u_{l}\right)}{\partial \xi^{j}}+\frac{1}{\rho_{0}}\left[\frac{\partial }{\partial \xi^{j}}\left(\mu \frac{g^{jk}}{J}\frac{\partial u_{l}}{\partial \xi^{k}}\right)-\frac{\partial }{\partial \xi^{j}}\left(\frac{\xi_{l}^{j}p}{J}\right)-\frac{\partial \tau_{lj}}{\partial \xi^{j}}+\left(\bar{\rho }-\rho_{0}\right)g\left(\frac{\delta_{i3}}{J}\right)\right]+f_{l}\right\}$$

where ξ/ represent the transformation metrics, J denotes the Jacobian of the transformation, U^j^ stands for the contravariant volume flux, $u_{I}$ denotes the Cartesian velocity component, p represents the pressure, $\tau_{/j}$ signifies the Reynolds stress tensor for the LES model, $\delta_{ij}$ refers to the Kronecker delta, μ denotes the dynamic viscosity, g represents the gravitational acceleration, $\rho_{0}$ stands for the background density (which in our case is the density of air), $\overset{‾}{\rho }$ represents the density of the air–saliva mixture, and $f_{I}$ (where I=1,2,and3) symbolizes the body force introduced by the facial mask. The dynamic Smagorinsky model is utilized to characterize the sub-grid scale (SGS) terms of the LES.^70^.

### 2.2. Saliva plume transport

The interaction between air and saliva within the mixture causes fluctuations in the density of the air-saliva mixture, which, in turn, impacts the Eulerian fluid motion. These density variations, which are determined using the Boussinesq assumption, are the following^71^:

(3) $$\rho =\rho_{0}(1-\psi )+\rho_{s}\psi$$

where ψ and $\rho_{s}$ denote the volume fraction and density ($1000\;kg/m^{3}$) of the saliva particles in the mixture, respectively. The following convection-diffusion equation governs the saliva particles’ concentration^71^:

(4) $$\frac{1}{J}\frac{\partial \left(\rho_{0}\psi \right)}{\partial t}=\frac{\partial }{\partial \xi^{j}}\left[\left(\mu \sigma_{L}+\mu_{t}\sigma_{T}\right)\frac{g^{jk}}{J}\frac{\partial \psi }{\partial \xi^{k}}\right]-\frac{\partial }{\partial \xi^{j}}\left[\rho_{0}\psi \left(U^{j}-W^{j}\delta_{i3}\right)\right]$$

where the vertical contravariant volume flux $W_{j}$ of saliva concentration, induced by the particles’ settling velocity $w_{s}$, is expressed as $\left(=\frac{\xi_{3}^{j}}{J}w_{s}\right)$. Here, $\mu_{t}$ represents the eddy viscosity, while $\sigma_{L}$ and $\sigma_{T}$ respectively denote the laminar and turbulent Schmidt numbers, assigned values of 100 and 0.75^72^. The settling velocity of the particles is determined as such^60^:

(5) $$w_{s}=\frac{D_{p}^{2}\left(\rho_{s}-\rho_{0}\right)}{18\mu }$$

where $D_{p}$ is the diameter of the saliva particle.

### 2.3. Evaporation effect

**Figure 1:**
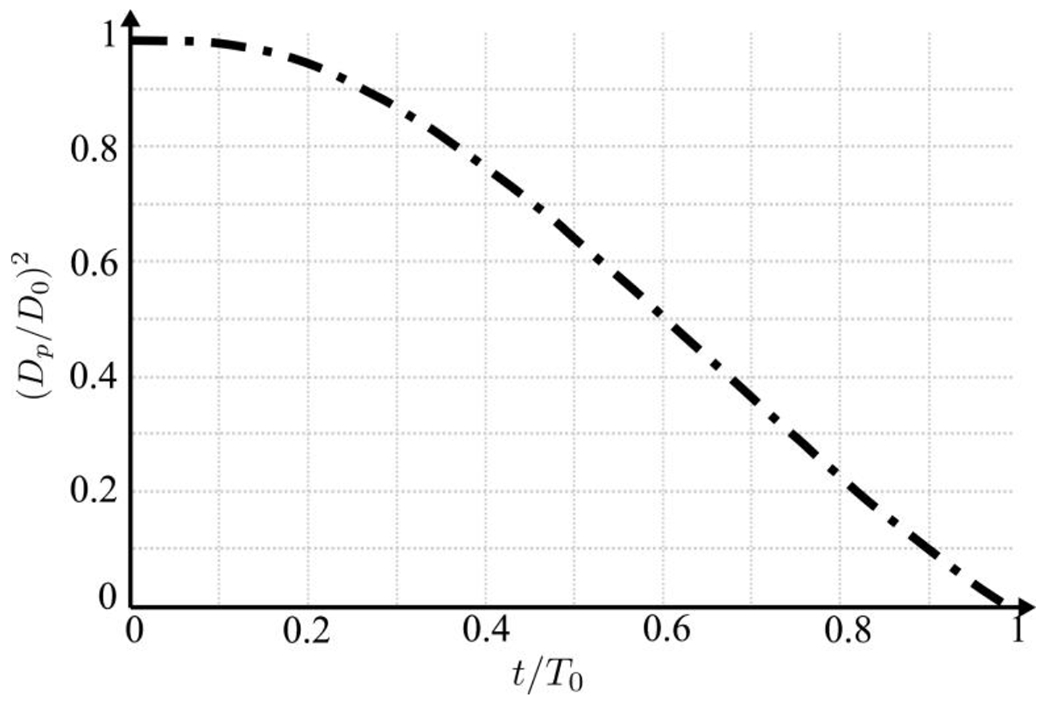
The influence of evaporation on saliva particles is depicted by the time evolution of the square of the normalized saliva particles’ diameter. Beyond $t/T_{0}=1$, the ratio ${\left(D_{p}/D_{0}\right)}^{2}$ stabilizes at $10^{-4}$.

Evaporation exercises a notable influence on saliva plumes’ dispersion dynamics, which is extensively documented in scientific literature^10,21,31,73–75^. Even so, quantifying the precise rate of saliva evaporation remains challenging because of its dependence on ambient conditions such as humidity, temperature, and the velocity of saliva particulates in the surrounding air. To address this, we adopt an empirical approach wherein we systematically reduce the exhaled saliva particles’ size, thereby modulating their settling velocity. This strategy is motivated by previous research. Notably, the work of Mittal et al.^21^ observed rapid evaporation of exhaled saliva particles and a subsequent decrease in size. In our computational framework, we integrate this reduction rate based on empirical data reported by Li et al.^73^, which delineates the temporal evolution of saliva particle size. Initially, the mixture of exhaled air and saliva, characterized by 10 *μm* saliva particles, undergoes swift evaporation upon encountering stagnant ambient air, resulting in a reduction in size to 0.1 *μm* within a time span of 0.2 s. Subsequently, the particle size stabilizes at 0.1 *μm*. The temporal variation of ${\left(D_{p}/D_{0}\right)}^{2}$ is represented nonlinearly in Figure 1 to model particle evaporation. Here, $D_{p}$ denotes the particle diameter, normalized by the initial diameter $D_{0}=10\;\mu m$. In line with prior studies^76^, larger droplets typically greater than 5 *μm* in diameter tend to remain lodged in the upper respiratory tract, particularly in the oropharynx (nose and throat regions). Consequently, it is inferred that saliva particles emitted from the nose and mouth share a similar size range. Moreover, it is postulated that saliva particles stabilize at a diameter of 0.1 *μm* shortly after exhalation and maintain this size thereafter. This stabilization occurs roughly 0.2 s after exhalation, denoted as $T_{0}=0.2\;s$. Once the water content evaporates from a saliva droplet, it settles at a stable final size, which persists for hours. Across droplets of varied sizes, researchers have observed a consistent pattern wherein the final diameter aligns with approximately 20% of the initial diameter. This correlation remains steady regardless of ambient conditions, ranging in temperature from 20°C to 29°C. The presence of salts and proteins within saliva droplets contributes to this phenomenon^77^.

### 2.4. LAGRANGIAN PARTICLE TRACKING

We employ a Lagrangian approach for tracking individual saliva particles, which involves one-way coupling. Initially, we solve the Eulerian equations governing the flow of the air-saliva mixture (Eqs. 1–5). Next, we utilize a Lagrangian particle tracking technique to compute individual saliva particles’ paths within the Eulerian flow field. Focusing on a dilute mixture of air and saliva, we address the following equations for each saliva particle^60^:

(6) $$\frac{\partial x_{p}}{\partial t}=u_{p}$$

(7) $$m_{p}\frac{\partial u_{p}}{\partial t}=f_{g}+f_{d}+f_{l}+f_{am}+f_{s}+f_{p}$$

where $x_{p}$ and $u_{p}$ respectively represent the particle’s position and velocity, $m_{p}$ denotes the particle’s mass, $f_{g}$ denotes the gravity force, $f_{d}$ denotes the drag force, $f_{l}$ denotes the lift force, $f_{am}$ denotes the added mass force, $f_{s}$ denotes the collision forces from solid boundaries on the particle, and $f_{p}$ denotes the force due to fluid stresses. In this study, we only consider the gravity and drag forces^60^. The drag coefficient depends on the given range of the Reynolds number^78^:

(8) $$C_{d}=\{\begin{array}{cc} \frac{24}{Re_{p}}, & Re_{p}<0.1\\ 3.69+\frac{22.73}{Re_{p}}+\frac{0.0903}{R{e_{p}}^{2}}, & 0.1<Re_{p}<1\\ 1.222+\frac{1.667}{Re_{p}}-\frac{3.889}{R{e_{p}}^{2}}, & 1<Re_{p}<10 \end{array}$$

where $Re_{p}=\rho_{0}D_{p}∥\left(u_{f}-u_{p}\right)∥/\mu$ is the saliva particles’ Reynolds number. Given the particle sizes and flow velocities detailed in section 4, *Re_p_* consistently remains below 10.

### 2.5. BOUNDARY CONDITIONS

Throughout the simulations, the boundary conditions comprise a no-slip boundary condition on the floor and on the human anatomy, a periodic boundary condition in the spanwise direction, and an outflow Neumann-type boundary at the outlet and top. The subsequent expression calculates the inlet velocity of normal breathing:

(9) $$u=\frac{V_{t}}{T_{b}\cdot A}\pi \;\cos\left(\frac{2\pi t}{T_{b}}+\frac{\pi }{2}\right)$$

where $V_{t}$ represents the tidal volume of breathing $\left(1\times 10^{-3}m^{3}\right),T_{b}=5\;s$ denotes the breathing period, t indicates the instantaneous time, and A represents the area of the outlet opening. This equation yields a maximum velocity of approximately 0.9m/s. Given that 75% of the tidal volume is expected to pass through the mouth and 25% through the nose, we assign a horizontal velocity component of 0.75*u* at the mouth while setting the exit velocity of the air–saliva mixture from the nose to be 0.25*u* in magnitude^14,60^.

## 3. Human anatomy and face mask geometry

**Figure 2:**
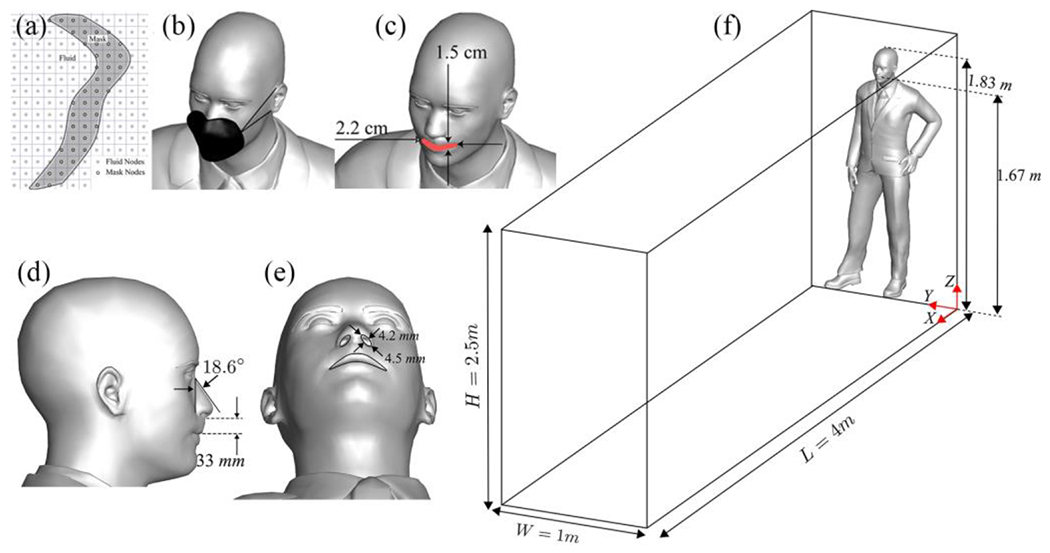
Schematic of the simulation domain, human anatomy, and the face mask, (a) Grid system illustrating the background grid, face mask, and human anatomy in immersed boundary method, (b) face mask with a thickness of 2 mm, (c, d, e) opening of the nose and mouth, and (f) computational domain and human anatomy.

For an accurate representation of the human body’s complex three-dimensional structure, we use the immersed boundary method^14,43^ (see Figure 2). To model the face masks’ effects, we employ a diffused-interface immersed boundary technique that applies a drag force to the unstructured triangular grid nodes responsible for discretizing the mask’s three-dimensional geometry, as shown in Figure 2-a and b^14^. The facial mask’s drag force is transferred to the fluid nodes using a smoothed discrete delta function. This distribution mechanism is expressed as^43^:

(10) $$f_{l}=\frac{1}{2}\bar{\rho }C_{d}A{\left(u_{i}u_{i}\right)}^{1/2}u_{l}\delta \left(x_{j}-X_{j}\right)$$

where *C_d_* represents the drag coefficient, *A* denotes the face mask’s projected area, *u_i_* refers to the local Cartesian velocity vector, and δ stands for the smoothed discrete delta function.

The nose and mouth’s geometry, a crucial aspect of our simulations, is visualized in Figure 2-c, d, and e. The face mask exhibits asymmetrical curvatures around the face, with varying thickness at different locations and an average thickness of 2 mm, as depicted in Figure 2-a and Figure 2-b. According to prior research, a drag coefficient of 350 is employed for a standard non-medical grade mask, which is employed in this study^37^. The human anatomy and mask geometry are generated using Blender, an open-source software tool.

## 4. Computational details

We investigate two sets of scenarios: coughing and normal breathing. For each scenario, simulations are conducted with and without the presence of a face mask to assess its impact. Specifically, simulations examine indoor stagnant air and outdoor airflow conditions for coughing scenarios. In coughing events, breathing originates solely from the mouth. In contrast, normal breathing scenarios implement mouth-only breathing and combined nose and mouth breathing to explore the influence of nasal airflow dynamics. The simulation is conducted within a computational domain measuring 4.0 m in length, 1.0 m in width, and 2.5 m in height. The computational grid’s spatial resolution is set at 0.5 mm near the nose and mouth with a stretching ratio of 1.002 in all directions, and the temporal resolution is set at 0.5 ms to yield a Courant–Friedrichs–Lewy (CFL) number less than 1. This setup yields over 650 million computational grid nodes.

The human geometry created for the simulation stands at a height of 1.82 m, with the mouth positioned at 1.67 m above ground level, with dimensions of 0.022 m width and 0.015 m height. The lower part of the nose sits 0.033 m higher than the mouth. Inclined at an angle of 18.6 degrees relative to the vertical direction (z-axis), the nose exhibits a curved elliptical shape for its opening, measuring approximately 0.0351 m in width and 0.01 m in height. The nose opening consists of two ellipses, each featuring a standard nostril width of 0.009 m. These ellipses’ major and minor axes are 0.0045 m and 0.004265 m, respectively. Notably, the nostrils’ minor axis is inclined at an angle corresponding to the nose’s orientation concerning the z-direction. This geometric configuration provides a realistic representation of the nose, facilitating the investigation of various fluid-dynamic factors affecting normal human breathing. Lagrangian particle tracking is performed for 110,000 saliva particles released from the nose and mouth openings into the room.

The simulation effectively captures nearly all turbulent scales, as evidenced by the infrequent activation of the subgrid-scale model in the LES. The ratio between the computational grid size and the Kolmogorov scale verifies this. For example, the maximum Reynolds number during normal breathing is approximately *Re* = 600, characterized by a maximum velocity of 0.89 m/s and a characteristic length based on the mouth opening of L = 0.01 m. Then again, the highest Reynolds number is confined to a small region near the mouth, while the Reynolds number is considerably lower elsewhere throughout the simulations. Therefore, the Kolmogorov scale (*η*) of the smallest eddy in the breathing flow can be estimated by the relation $\frac{\eta }{L}∼e^{-3/4}$, resulting in approximately 8 × 10^−5^*m*. With a grid resolution of 0.5 mm near the mouth, the grid size ratio to the Kolmogorov length scale is approximately 6. According to Pope^79^, nearly all relevant scales of turbulence are resolved at this ratio. Therefore, this study is effectively a DNS of saliva-air mixture flow for indoor normal breathing.

The simulations of each case use 320 processors on a Linux cluster comprising 1216 Intel Xeon 3.3GHz cores, spanning nearly seven months of clock time. The message-passing interface (MPI) communication standard parallelizes the numerical model and operates on a parallel high-performance supercomputer. To streamline computational costs, the simulations make several simplifying assumptions: (a) the human model’s facial and anatomical features remain stationary throughout the breathing process; (b) stagnant air serves as the initial airflow conditions within the room; (c) the thermal stratification surrounding the body is disregarded; (d) condensation of exhaled saliva particles is omitted; (e) the density of exhaled particles remains constant throughout breathing; (f) saliva particles expelled from the nose and mouth fall within the same size range; (g) saliva particles are assumed to evaporate and stabilize at a diameter of 0.1 *μm*; (h) a mouth-to-nose breathing ratio of 75%–25% is considered, presuming a predominant exhalation through the mouth; (i) nasal airflow occurs at an angle of 18.6°; and (j) identical geometric configurations are assumed for both nasal openings. Nonetheless, further investigations are warranted to explore normal breathing patterns under varied mouth-to-nose breathing ratios and nasal angles.

## 5. Results and Discussions

Herein, we present the results of our high-fidelity numerical simulations for human respiratory events with and without face masks. The simulations were continued until the particles reached an equilibrium, where their momentum approached machine zero (i.e., 10^−8^). Each breathing cycle spans 5 s, with 2.5 s allotted respectively for inhalation and exhalation. During the cycle, saliva particles initiate evaporation upon exhalation, ultimately attaining a final size of 0.1 *μm* within a relatively short time frame of 0.2 s. The corresponding settling speed for these saliva particles is 0.3 *μm*/s, which requires several days to descend to the ground under stagnant ambient air conditions.

### 5.1. EULERIAN TRANSPORT OF SALIVA PLUME DURING COUGHING

**Figure 3:**
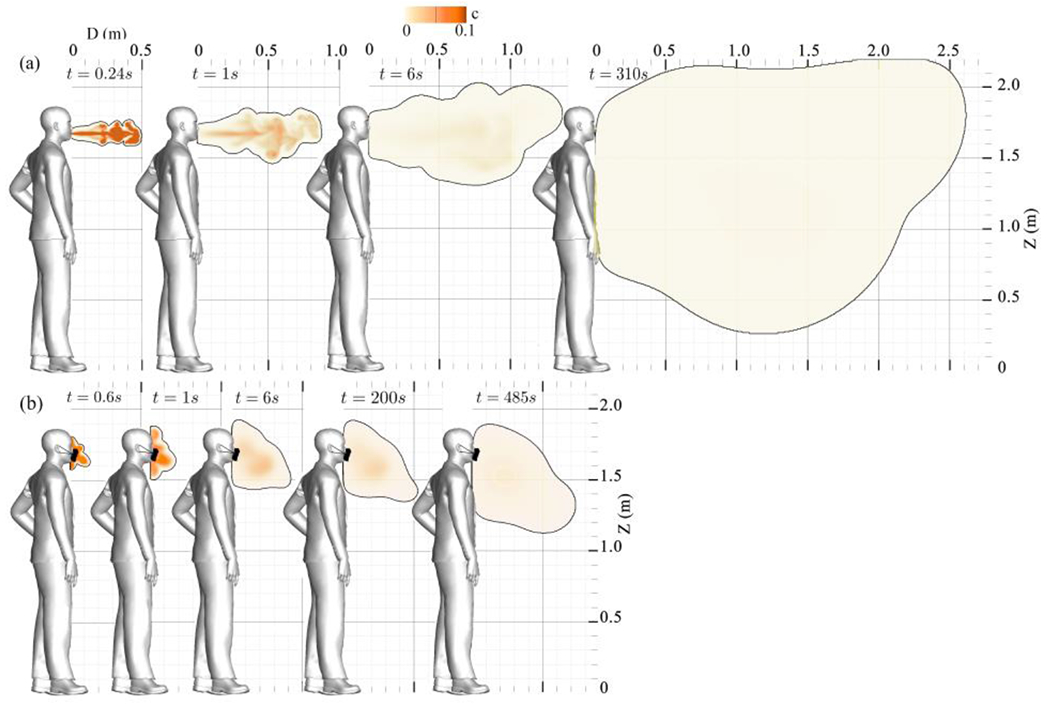
Saliva particle concentration (Volume Fraction) contours in an indoor coughing scenario for 10 *μm* particles on a sagittal plane. (a) without a face mask, and (b) with a non-medical type face mask. Results presented at (a) *t* =0.24, 1, 6, and 310 s, and (b) *t* =0.6, 1, 6, 200, and 485 s of simulation.

Figure 3 plots the instantaneous simulation results of an indoor cough scenario without a facial mask (a) and using a non-medical face mask (b) for particle sizes of 10 *μm*.

The rationale behind selecting 10 *μm* particles is their propensity to travel the greatest distance from the individual during a cough. Despite comprising less than 5% of the coughed saliva, these particles are of considerable size, and so are more capable of SARSCoV-2 virus transmission. The simulation results for indoor coughing without a face mask are significant for two main reasons. Firstly, they are crucial for individuals in the same room as the coughing person, as saliva particles can reach a streamwise distance of 2.62 m and a spanwise distance of 0.94 m concerning the respiration source. Secondly, these findings mark the effectiveness of face masks in reducing the dispersion of saliva particle plumes.

To demonstrate the impact of saliva particle evaporation, we conducted a simulation in which we initially ran the model using 10 *μm* particles for 120 s. Subsequently, we utilized the concentration field results of the saliva and continued the simulation with particle sizes reduced to 5.5 *μm*. This adjustment was necessary because saliva evaporation decreases particle size and settling velocity. The simulation continued until the particle plume reached near zero momentum (*t* = 310 s). The results revealed that the evaporated particles traveled approximately 2.84 m away from the source, indicating an 8% increase in traveling distance compared to the case with a constant particle size of 10 *μm*. Thus, evaporation effects can significantly affect particle travel distance.

**Figure 4:**
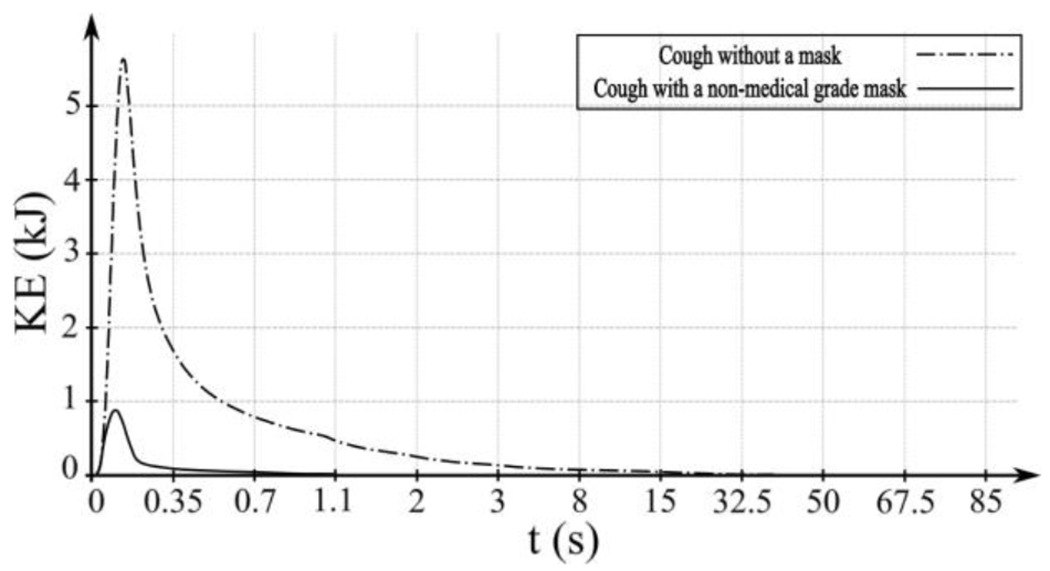
Comparative analysis of total kinetic energy-*KE* (*kj*) of saliva particles during coughing over time: unmasked vs. non-medical face mask. Noticeable reduction in total KE with face mask usage.

To examine the face mask’s effect in an indoor setting, in Figure 3-b, we present the instantaneous concentration results of masked coughed saliva particles with a size of 10 *μm*. In indoor coughing, the face mask effectively limits the spread of saliva particles by serving as a momentum sink. Additionally, the face mask seems to redirect the propagation of the cough plume vertically downward by dissipating its forward momentum (Figure 3-b). In addition, the face mask proves effective in dissipating the kinetic energy of the cough, reducing it by approximately one order of magnitude (Figure 4).

**Figure 5:**
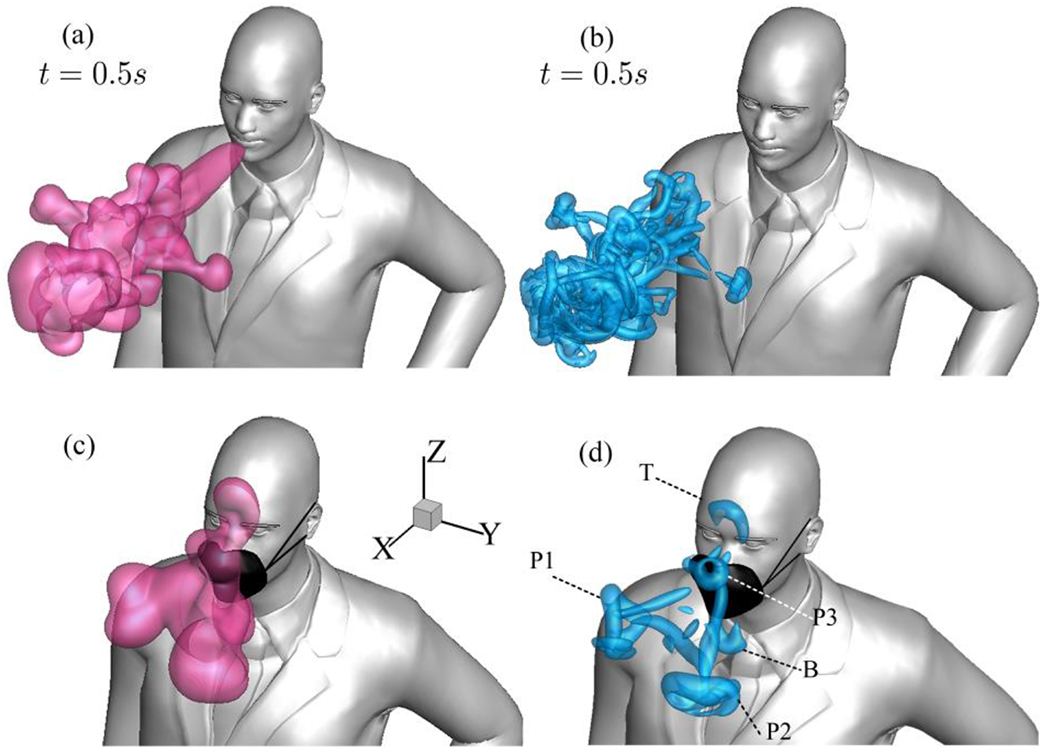
Visualization of three-dimensional coughing vortical structures emerging in turbulent cough jet at *t* =0.5 s: no mask (a and b), non-medical type face mask (c and d), utilizing iso-surfaces of saliva particle concentration (a and c) and iso-surfaces of Q-criterion (b and d).

Another set of results that demonstrate the efficacy of face masks involves the visualization of iso-surfaces of the Q-criterion. As depicted in the Figure 5, vortices labeled as B and T indicate the redirection of cough particles towards the bottom and top, respectively.

As seen by the analysis of the iso-surfaces (see Figure 5), vortices P1, P2, and P3 illustrate particle plumes that penetrate the face mask and move forwards. A comparative analysis between Figure 5-d and Figure 5-b reveals the advantage of mask-wearing in the forward spreading of particle plume during cough. To demonstrate the impact of saliva particle evaporation on masked coughing, we conducted another simulation in which we simulated 10 *μm* particles for *t* = 120 s, followed by a continuation of the simulation with a decrease in particle size to 5.5 *μm*. After 432 s, when the forward motion of the plume was stopped, we observed that saliva particles traveled a distance of 0.91 m away from the person, representing a 20% increase in traveling distance.

**Figure 6:**
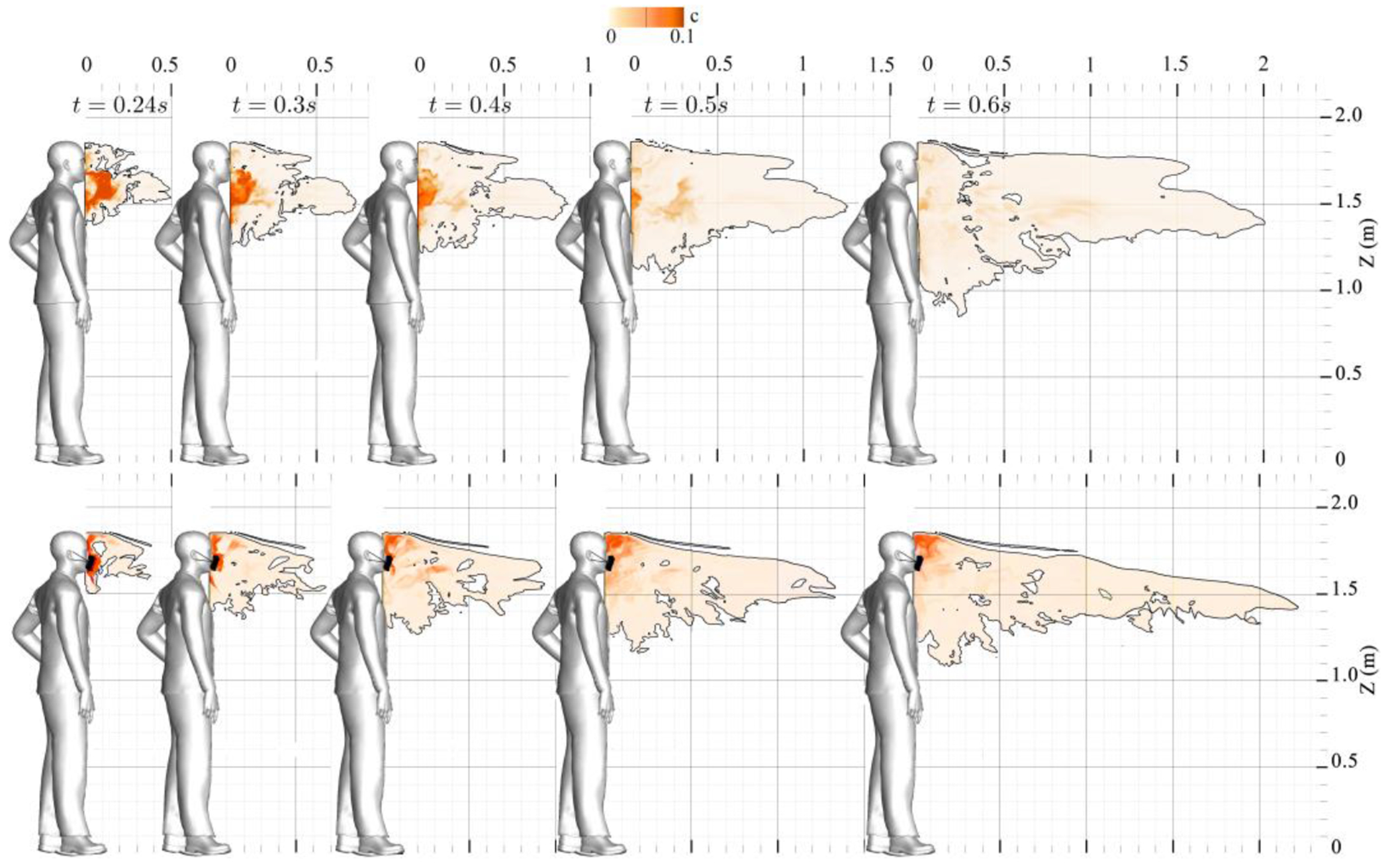
Saliva particle concentration (volume fraction) contours in an outdoor coughing scenario using a face mask on a sagittal plane. (a) is the no-mask case, and (b) is the non-medical type face mask case. Results presented at *t* =0.24, 0.3, 0.4, 0.5 and 0.6 s of simulation.

Since outdoor flow conditions might give rise to different background turbulence, we implemented a turbulent background flow condition resembling a unidirectional mild breeze with a mean flow velocity of 4.5 m/s during the coughing process with and without a face mask. Initially, the mild breeze was simulated, leading to a wake and a shear layer around the human body. Eventually, it evolved into a fully developed turbulent flow with a 3D energetic shear layer and reached statistical equilibrium. Subsequently, we modeled the coughing process and corresponding saliva transport. Figure 6 presents the instantaneous saliva particle concentration field with and without the face mask under outdoor breeze conditions. For the outdoor cough scenario, as depicted in Figure 6-a, Saliva particles are initially entrapped within the recirculation flow region in front of the face. However, soon after, particles escape from this region. Propelled by the background turbulent flow, particle plumes of the masked and unmasked cough propagate considerable distances, nearly reaching 2.0 m in less than a second. Moreover, in the case of wearing a face mask, as depicted in Figure 6-b, the transport process of saliva particle plume is approximately identical to the scenario without a face mask. This is mainly because face masks redirect saliva plumes upwards and downwards. Unlike indoor conditions, the turbulent background flow of the outdoor conditions rapidly transports these redirected particles, allowing them to travel nearly 2.2 m ahead of the coughing person in less than a second. Whereas the usage of face masks effectively curtails particle transport in indoor settings, it does not impact the transport of saliva particles in outdoor settings.

### 5.2. EULERIAN TRANSPORT OF SALIVA PLUME DURING NORMAL BREATHING

**Figure 7:**
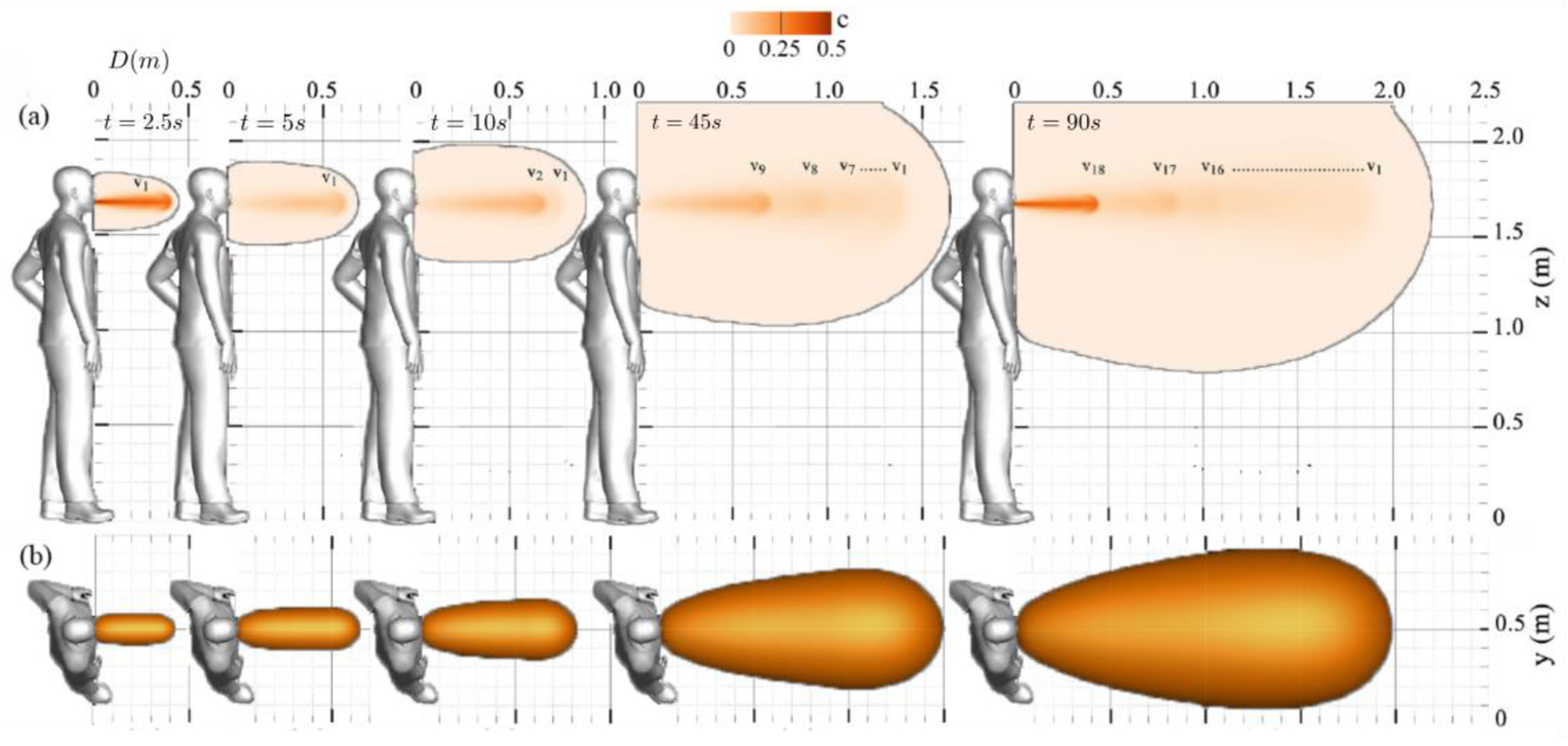
Saliva particle concentration (volume fraction) during normal breathing without a face mask: (a) contours on a sagittal plane reveal periodic leading vortex rings, denoted by *v_i_*, (b) iso-surface depiction of the concentration field on a top plane.

This section presents the Eulerian results of normal breathing for a range of saliva particle sizes, from 0.1 to 10 *μm*, to investigate their propagation with and without face masks. Figure 7 illustrates the saliva plumes’ instantaneous transport during normal breathing without a face mask, observed after 90 s of breathing when the saliva plume reaches equilibrium, i.e., when total flow momentum approaches zero. As seen, a single exhale possesses sufficient momentum to propagate up to 0.75 m away from the individual breathing (at *t* = 5 and 10 s). Consequently, the exhalation process accumulates momentum near the saliva plume’s leading edge, which drives the plume’s forward motion.

The accumulation of momentum from subsequent breathing cycles contributes to a rise in kinetic energy, driven by vortex rings’ successive generation with each breathing cycle. Alongside this overall energy escalation, a periodic surge results from inhale/exhale cycles. Conspicuously, the saliva plume can extend up to 2.2 m away from the individual after 18 breathing cycles (*t* = 90 s). Within 57 s, the plume reaches 1.8 m, which is the suggested social distancing according to the CDC. Furthermore, the temporal evolution of saliva particle size due to evaporation indicates that most saliva particles are approximately 0.1 *μm* in size Figure 1. These particles possess a settling velocity of 3×10^−7^ *m/s*. The particle plume may take days to settle into the ground. Consequently, the saliva plumes’ propagation and the saliva particles’ prolonged settling time underscore the need to revise regulations provided by the WHO and CDC.

A novel expansion of the analysis is the identification of coherent structures. The expelled air-saliva mixture manifests as a longitudinal jet-like flow, characterized by a shear layer that evolves into a leading circular vortex ring and a trailing jet (see Figure 7). To better explain this process, the leading vortex ring of the i-th breathing cycle is denoted as *V_i_* in this figure. Specifically, as depicted in Figure 7-a (*t* = 10 s), the leading vortex ring advances forwards until it separates from its trailing jet, a process resulting in the detachment of the vortex ring *v*_1_ from its original trailing jet.

**Figure 8:**
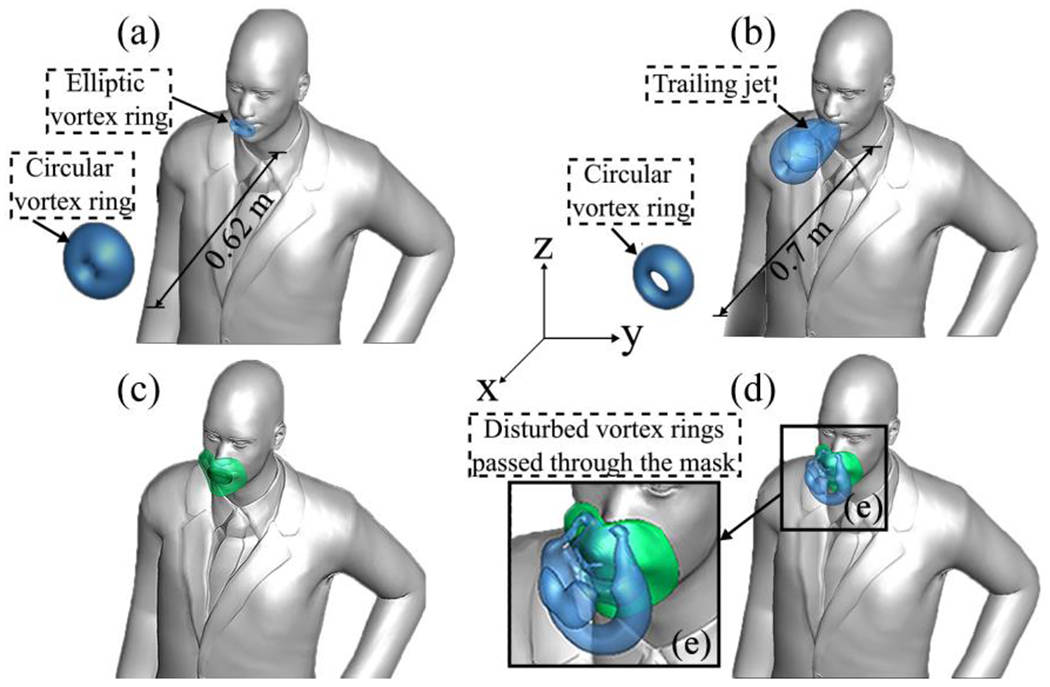
Evolution of vortical structure in normal breathing, visualized by iso-surfaces of Q-criterion (=0.1): (a) and (b) without a face mask, (c) and (d) with a non-medical type face mask. The simulated vortical structures for (a) and (c) correspond to *t* = 45.1 s, and for (b) and (d) to *t* = 61.2 s. Subparts of the vortical structures are apparent in each figure, showcasing the face mask’s effect by redirecting the vortical structures.

To enhance the visualization of vortical structures, we present iso-surfaces of the Q-criteria at two different time instances: *t* = 45.1 s and *t* = 61.2 s (Figure 8-a and b, respectively). From what we observed in Figure 8, based on looking into the iso-surfaces, as the vortex ring progresses downstream, it transitions into an axisymmetric circular vortex ring at a distance of 0.62 m downstream. In Figure 8-a, corresponding to 0.1 s after exhalation (*t* = 45.1 s), an elliptical vortex ring forms, resembling the mouth’s geometry. In Figure 8-b, corresponding to the moment when the velocity of the air-saliva mixture exiting the mouth reaches its peak, a trailing jet is observed connected to a leading vortex ring. This leading vortex ring experiences a pinch-off process 0.7 m downstream from the mouth. Hence, the leading vortex ring’s coherent structure significantly influences the dynamics of particle plume transport.

Following the initial phase of normal breathing, as breathing continues, newly formed vortex rings propagate forwards and intersect with previously generated rings (see Figure 7). This ongoing process gradually decelerates the saliva plume until it reaches a state of equilibrium.

**Figure 9:**
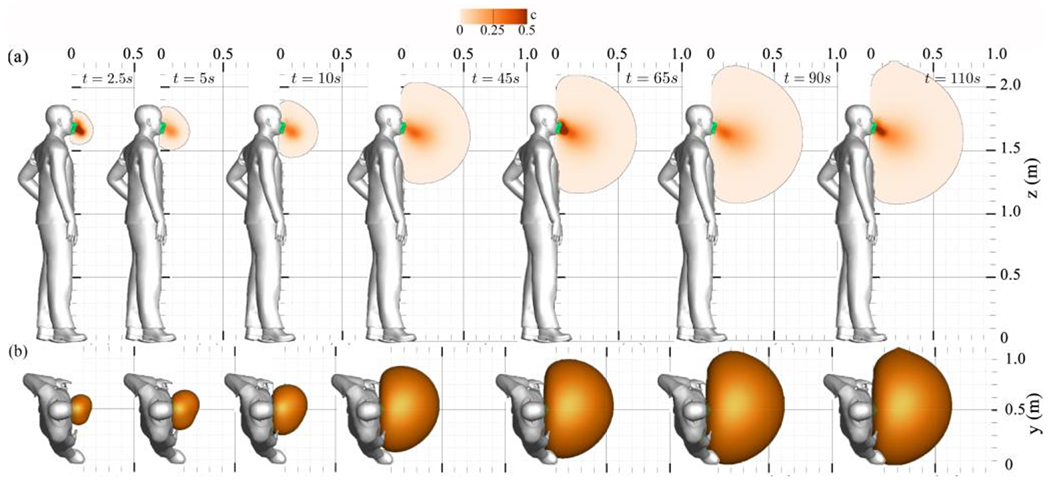
Saliva particle concentration (volume fraction) during normal breathing with a non-medical type face mask is depicted in the following figures: (a) contours on a sagittal plane, and (b) Iso-surface depiction of the concentration field on a top plane. The asymmetrical distribution is attributed to the heterogeneity of the face mask’s thickness.

Figure 9 depicts the instantaneous saliva plume results for the normal breathing scenario with a face mask.

**Figure 10:**
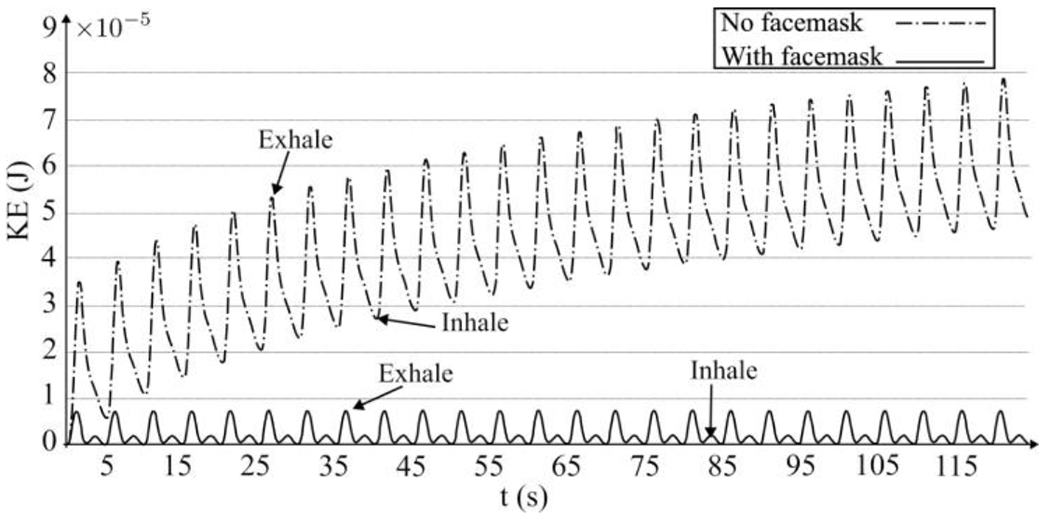
The total kinetic energy over time for normal breathing scenarios, comparing without a face mask (depicted by the dotted-dashed line) and with a non-medical face mask (represented by the solid line). The decreasing trend in total kinetic energy demonstrates the efficacy of the face mask in mitigating kinetic energy levels.

As seen for the masked coughing in section 5.1, the face mask mitigates the forward momentum and redirects the breathing jets (Figure 9). This momentum reduction is primarily attributed to the dissipation of energetic vortical structures. Such reduction is evident in the analysis of time series of kinetic energy, illustrated in Figure 10.

Notably, when wearing a face mask, the total kinetic energy decreases by approximately one order of magnitude compared to the scenario without a mask. Examining the kinetic energy dynamics with a face mask reveals periodic fluctuations characterized by local peaks, with crests representing the exhale cycle and troughs representing the inhale cycle. Consequently, an overall increase in kinetic energy is not observed in this scenario.

**Figure 11:**
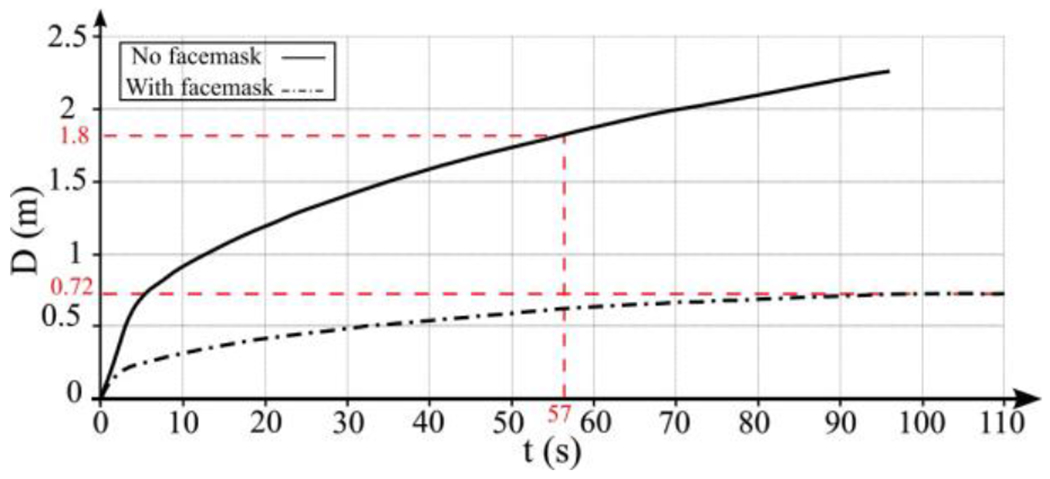
The length of saliva particle plume transport is compared between normal breathing with and without a face mask. Without a face mask (depicted by the straight line), the saliva particle plume propagates 1.8 m after *t* = 57 s. Conversely, with a face mask (illustrated by the dotted-dashed line), the saliva particle plume travels 0.72 m after *t* = 102.5 s, indicating the efficacy of the face mask.

The impact of face masks on normal breathing vortex dynamics is clearly illustrated in Figure 8-c and d. An elliptical vortex ring forms initially in the area between the mouth and the face mask, carrying sufficient kinetic energy to transport saliva particles downstream. However, upon passing through the face mask, this vortex ring dissipates shortly after reaching 0.15 m from the mouth. It undergoes deformation, affecting both the trailing jet and the leading vortex ring. Consequently, our findings indicate that wearing a face mask inhibits the formation of periodic trailing jets and leading vortex rings, thereby impeding the forward propagation of the saliva particle plume. These observations are corroborated by Figure 11, demonstrating that when using a face mask, the saliva plume’s travel distance is restricted to approximately 0.72 m after 102.5 s until reaching equilibrium. This distance falls well below the CDC’s recommended social distancing guideline of 1.8 m.

### 5.3. LAGRANGIAN TRANSPORT OF SALIVA PARTICLES DURING NORMAL BREATHING

We employed the coupled Eulerian-Lagrangian (EL) approach to map out the paths of saliva particles. To do so, we initially solved for the Eulerian air-saliva mixture flow. Subsequently, we trace the paths of individual saliva particles within this Eulerian flow field using a Lagrangian particle tracking approach. By analyzing these trajectories, we can identify LCSs, which represent the surfaces delineating the paths of the particles.

As particles evaporate, their Reynolds number rapidly decreases, transitioning to ambient laminar flow around them. Consequently, particles closely adhere to the flow field. This phenomenon is further elucidated in section 5.2, where we explore the air-saliva plume’s composition, highlighting a leading circular vortex ring and a trailing jet. As Lagrangian particles follow the Eulerian flow field, the saliva particles manifest remarkable fractal-like LCSs, comprising vortex rings and associated trailing structures periodically shed during each breathing cycle. Based on the definition of attracting and repelling manifolds^51^, the LCS obtained in our analysis visualizes the flow’s attracting and repelling regions. These regions represent areas where saliva particles cannot traverse the hyperbolic regions’ manifolds.

**Figure 12:**
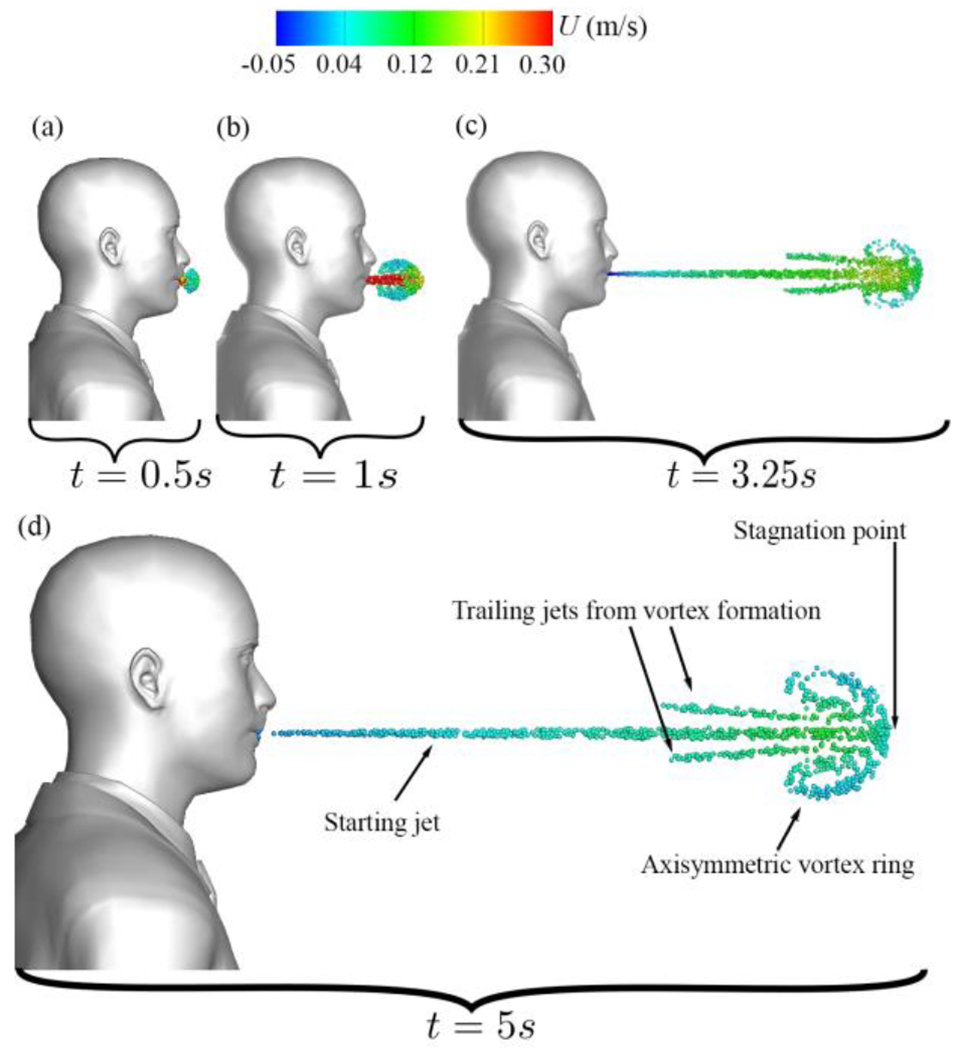
Evolution of coherent flow structures during initial breathing efflux through the mouth: (a) initial particle expansion at *t* = 0.5 s, (b) formation of the first vortex ring at *t* = 1 s, (c) development of trailing jets at *t* = 3.25 s, (d) coherent flow structures for mouth breathing. Particle colors indicate streamwise velocity (m/s) and are displayed on a 5 cm thick layer in the sagittal plane.

Figure 12 focuses on vortical structures identified by the trajectory surfaces of saliva particles during the initial stages of normal breathing through the mouth only. As seen in Figure 12-a, the particle cloud expands radially during the early stages of mouth breathing. Subsequently, the particle cloud undergoes roll-ups, forming a forward-propagating elliptic vortex ring that mirrors the shape of the mouth geometry (Figure 12-b). After 2.5 s, as the breathing cycle transitions into the inhalation phase, the propagated vortex ring undergoes a pinch-off process (Figure 12-c)^80^. At this juncture, some particles are drawn back into the mouth due to inhalation, contributing to the formation of vortical structures. These structures include a vortex ring accompanied by two axisymmetric trailing jets. This process results in a decrease in kinetic energy and an increase in generated thrust. As particles advance, they gradually lose momentum. By the conclusion of the first breathing cycle (at 5 s, Figure 12-d), they have traveled to a distance of 0.625 m away from the mouth, with a mean streamwise velocity of 5.42 cm/s.

**Figure 13:**
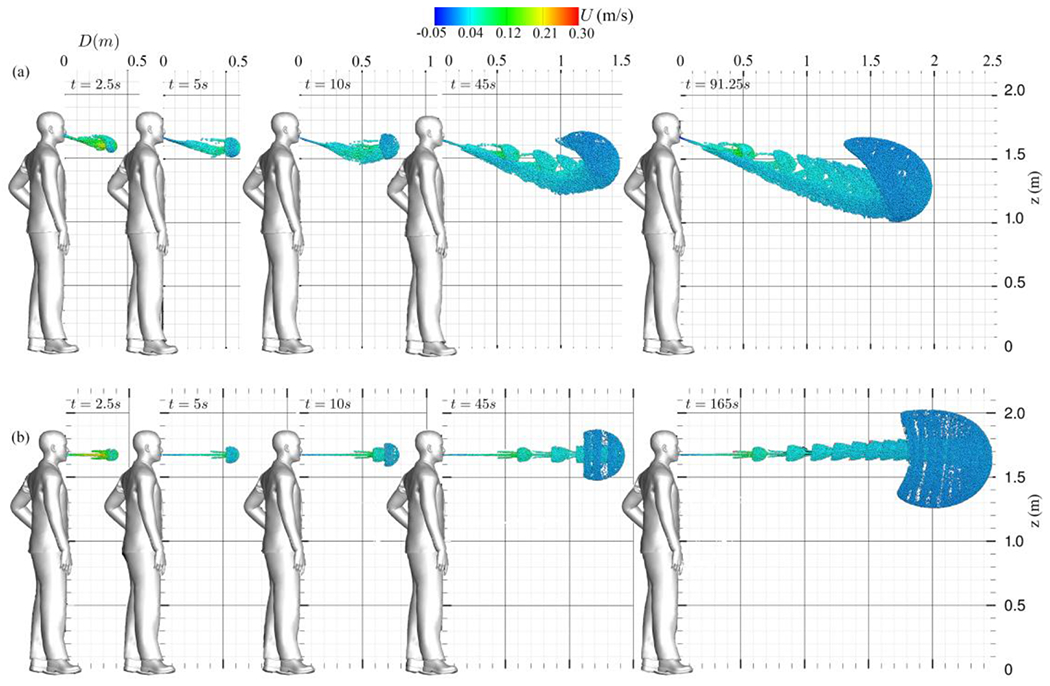
Simulation results of saliva particles transport evolution through time during normal breathing on a sagittal plane view: (a) nose and mouth^61^ and (b) mouth only^60^. Saliva particles are colored with their streamwise velocity (m/s).

The evolution of particle transport occurs by colliding newly exhaled particles with those from the initial cycle. Within collisions of particles from various cycles, momentum is transferred from the newly exhaled particles to the plume’s collective momentum. As a result, the plume radially expands and advances forward. Following the collisions, the particle plume assumes a spherical shape at the front, composed of particles from the most recent exhalations. This process persists as momentum transfer continues with subsequent collisions between newly exhaled particles and those from earlier cycles. This continual addition of momentum to the particle front propels it forward until the particles reach equilibrium, at which point their movement diminishes considerably (see Figure 13).

**Figure 14:**
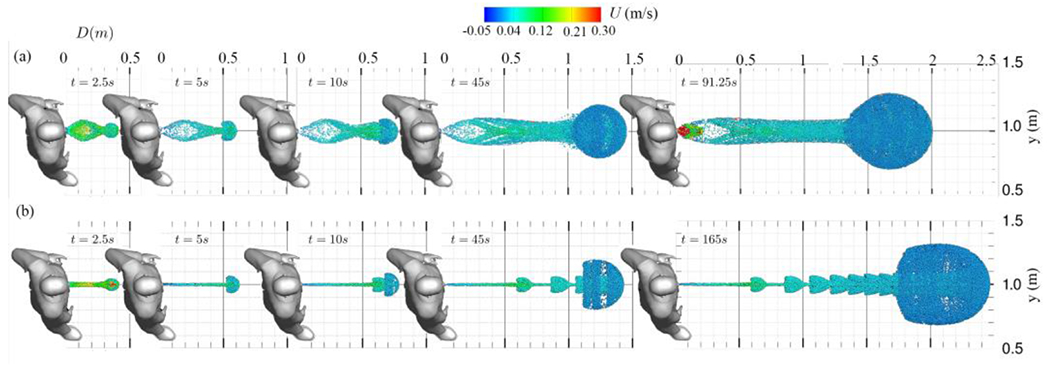
Simulation results of saliva particles transport evolution through time during normal breathing on a top plane view: (a) nose and mouth^61^ and (b) mouth only^60^. Saliva particles are colored with their streamwise velocity (m/s).

**Figure 15:**
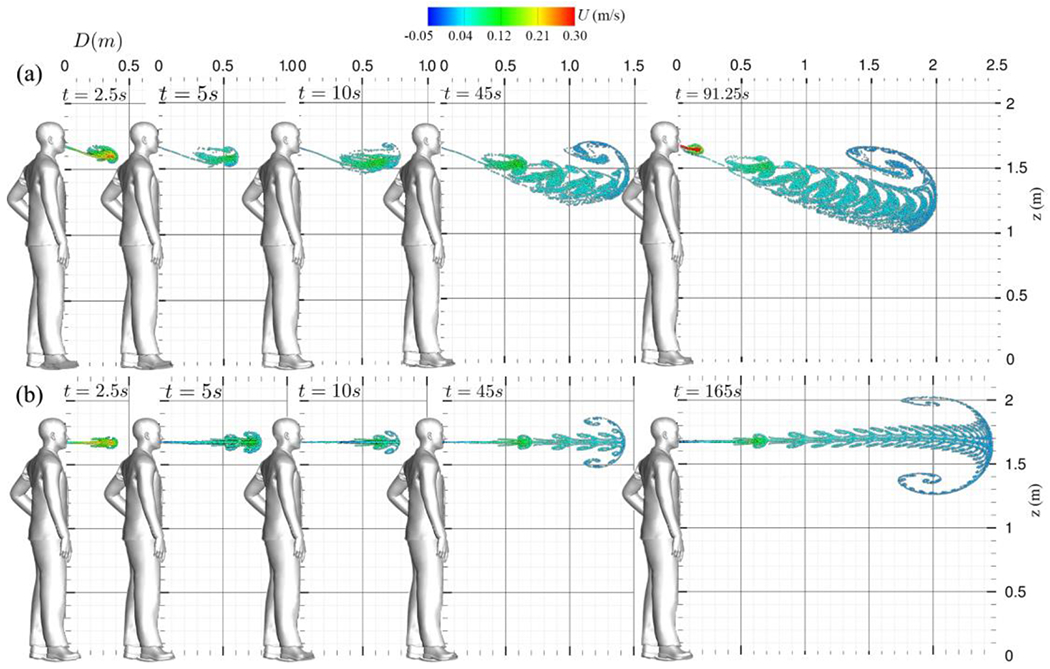
Simulation results of saliva particles transport evolution through time during normal breathing on a 5 cm thick sagittal plane view: (a) nose and mouth^61^ and (b) mouth only^60^. Saliva particles are colored with their streamwise velocity (m/s).

For a more comprehensive evaluation of saliva particles’ trajectories, we examine Figure 13 and Figure 14, which display the material surfaces in the 3D view from side and top views, respectively. Also, Figure 15 plots the material surfaces of saliva particles on a 5 cm thick sagittal plane. These figures plot a vortex ring and its trailing jets during each breathing cycle. The vortex ring accelerates due to the leading ring’s velocity, and it eventually captures and entrains the trailing jets. Over time, this vortex front decelerates, giving rise to fractal-like structures Figure 15. In addition, as seen in Figure 13 and Figure 15, a noticeable skewness towards the ground can be observed. The downward-tilted particle plume is due to the stratification effect, which is represented by the Boussinesq term in the momentum equation.

Figure 15-a also presents a Poincare map-like representation of particle transport through both the nose and mouth, compared to the mouth-only scenario (Figure 15-b). This depiction illustrates the trajectories of saliva particles within a 5 cm thick plane in sagittal view. As previously discussed, analysis of this visualization reveals fractal-like LCS, including vortex rings and trailing structures formed by the rapid expansion of the unsteady shear layer. Contrasting with the mouth only scenario (Figure 15-b), we observe the bending of the particle plume due to the nose’s crossflow effect on the mouth’s jet, along with the discernible roll-up of the vortex ring. In comparison to the mouth-only scenario (Figure 15-a), where a symmetrical plume with a circular forefront vortex containing upper and lower limbs is observed, the combined nose and mouth scenario exhibits alterations in the plume’s shape and the characteristics of the fractal-like LCSs.

**Figure 16:**
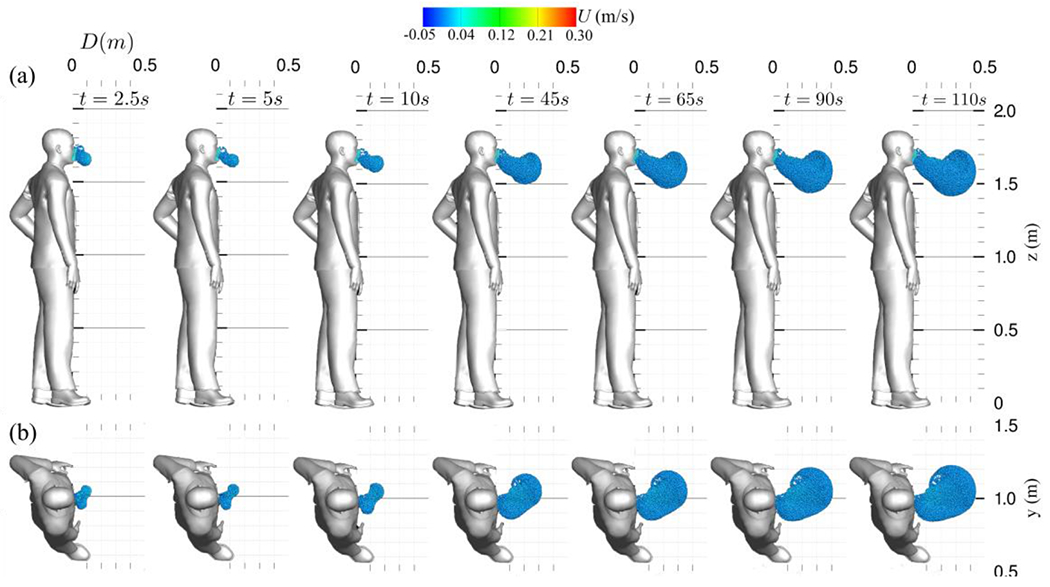
Simulation results showing saliva particle transport evolution during normal breathing with a non-medical grade face mask covering only the mouth: (a) sagittal view, and (b) top view. The face mask effect perceptibly alters the flow trajectory. Saliva particles are color-coded based on their streamwise velocity (m/s).

Figure 16 plots the instantaneous results of saliva particle transport during masked normal breathing. As discussed in sections 5.1 and 5.2, the face mask redirects saliva particles downwards and diminishes their forward momentum. In the Figure 16, it is observable that some particles become trapped in the space between the face and the mask. Having lost kinetic energy while passing through the mask, the remaining particles experience a reduction in momentum. Upon passing through the mask, these particles descend slowly due to gravity and the face mask’s asymmetrical shape and varying thickness, resulting in an asymmetric particle distribution. In addition to the momentum loss incurred by passing through the face mask, newly exhaled particles also impart a small amount of momentum to previously exhaled particles. Unlike the scenario without a face mask, there is no dominance of vortical structures. The maximum distance traveled by saliva particles during the masked breathing is 0.48 m at *t* = 142 s, with the highest velocity recorded at 1.5 mm/s. This distance is notably below the CDC social distancing regulation of 1.8 m, reiterating face masks’ efficacy in halting the forward propagation of virus-laden saliva particles.

**Figure 17:**
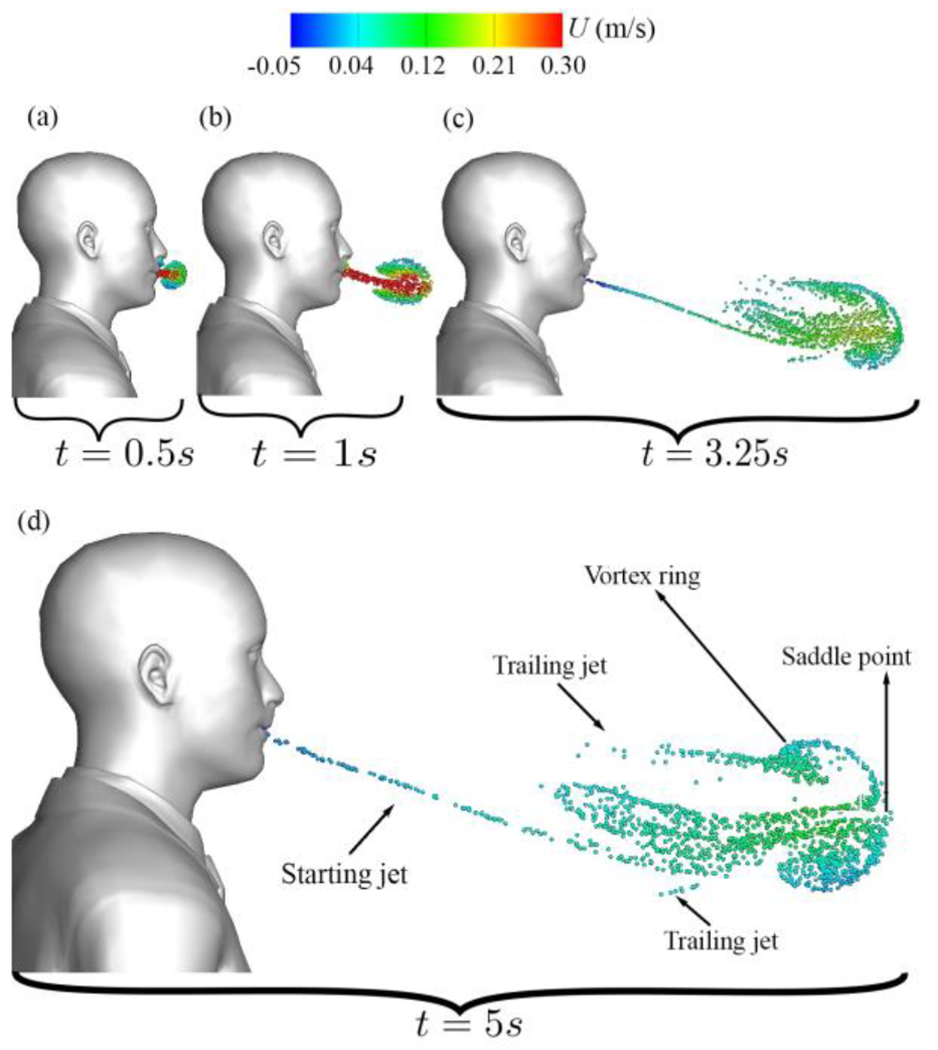
Evolution of coherent flow structures during initial breathing efflux through nose and mouth: (a) initial particle expansion at *t* = 0.5 s, (b) formation of the first vortex ring at *t* = 1 s, (c) development of trailing jets at *t* = 3.25 s, (d) coherent flow structures for nose and mouth breathing. Particle colors indicate streamwise velocity (m/s) and are displayed on a 5 cm thick layer in the sagittal plane.

Now we focus on normal breathing with mouth and nose, discussing the Lagrangian results of normal breathing through both the nose and mouth and juxtaposing them with the results of mouth-only breathing. As shown in Figure 17-a, at the onset of the first nose/mouth breathing cycle, the nose flow initially exerts no discernible influence on the particle cloud, resulting in a similar shape compared to mouth-only breathing in Figure 12-a).

However, soon after, as seen in Figure 17-b, the nose effect deflects the mouth jet downwards, marking a deviation from the mouth-only breathing scenario. As discussed in section 5.3, the subsequent inhalation process initiates the pinch-off phenomenon, culminating in the complete formation of the vortex ring and the concomitant emergence of two trailing jets. These trailing jets exhibit a symmetric behavior for mouth-only breathing (see Figure 12-c) while they exhibit an asymmetric configuration in the case of nose and mouth breathing (see Figure 12-c and Figure 17c). These dynamics were previously reported in^60^ and^61^. The asymmetry observed in the nose and mouth breathing scenario is attributed to the cross-flow effect that the nose exerts on the mouth jet’s flow. Ultimately, as the process progresses and the vortical structure forms towards the conclusion of the first breathing cycle, one can discern the vortical coherent structures for both nose and mouth breathing (see Figure 17-d) in contrast to mouth-only breathing (see Figure 12-d).

In the later stages of normal breathing through the nose and mouth, we observed the gradual accumulation of momentum over time and the formation of coherent flow structures, including vortex rings and trailing jets. Each vortex ring undergoes a roll-up process, contributing to the development of coherent structures. The nose/mouth breathing simulations are extended until the saliva particles reach an equilibrium state, which occurs after 91.25 s. At this point, the saliva plume has extended up to 2 m, surpassing the CDC social distancing guideline of 1.8 m.

**Figure 18:**
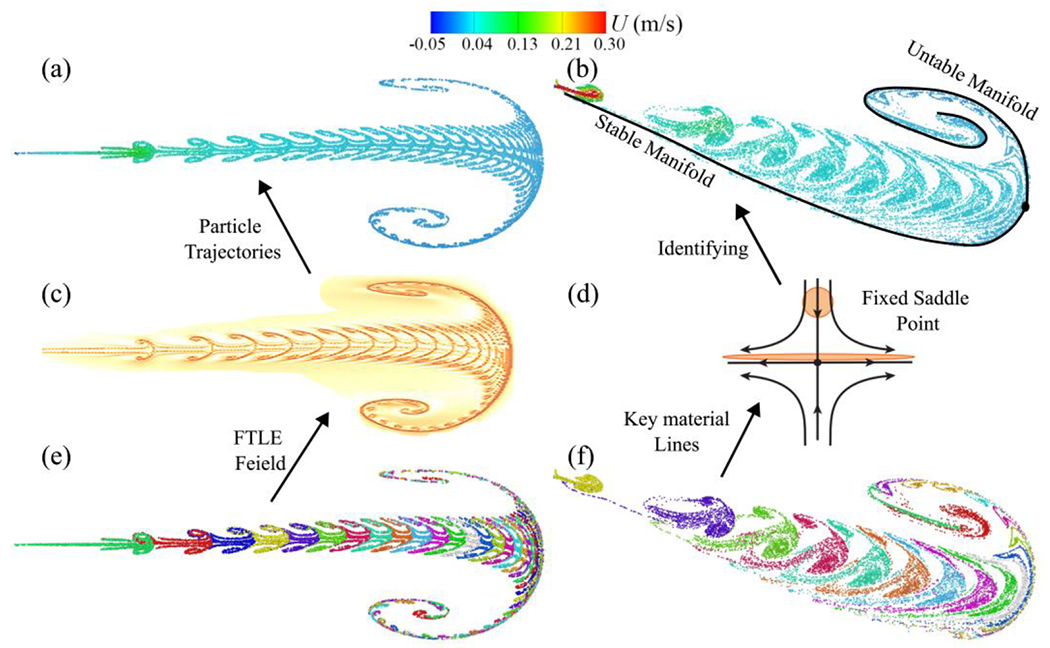
LCS diagnostics using (b) FTLE field and (d) identification of key material lines. Trajectories of saliva particles during normal breathing through the mouth only, colored by streamwise velocity (a), and breathing cycles (e); and normal breathing through both nose and mouth, colored by streamwise velocity (b), and breathing cycles (f). Saliva particle trajectories are visualized on a 5 cm sagittal plane for (a and e) mouth only at *t* = 165 s, and (b and f) nose and mouth at *t* = 91.25 s. Each color in (e and f) corresponds to a breathing cycle.

Moreover, LCSs delineate the material surfaces that organize fluid flow, playing a pivotal role in Lagrangian particle dynamics. These structures uncover the vortical patterns within the respiratory flow field. Therefore, we undertake the diagnosis of LCS generated by coupled EL simulations of normal breathing. In accordance with Haller^51^, LCS can be visualized either through Finite-Time Lyapunov Exponents (FTLE)^81^ or by identifying crucial material lines within the air-saliva mixture during normal breathing. For normal mouth-only breathing^60^, FTLE serves to detect invariant manifolds of hyperbolic LCSs intimately linked with the trajectories of saliva particles. To this end, LCS diagnostics employing FTLE run on a sagittal 2D plane (comprising streamwise and vertical components). Initially, the flow map *ψ* for normal breathing is computed through Eulerian LES. Subsequently, this flow map facilitates the determination of Green’s deformation tensor, from which the principal eigenvalue yields the FTLE field. Guided by these procedures, we plot the FTLE field calculated in backward time at *t* = 165 s (Figure 18-c), outlining the particle transport during normal breathing through the nose and mouth.

In our investigation of LCS diagnostics concerning normal human breathing via both the nasal and oral airways, we adopted an additional approach centered on identifying pivotal material lines^51^. The salient feature in Figure 18-b is the identification of the stable manifold delineated by the curved longitudinal jet. Saliva particles follow this stable manifold until they encounter the vortex front. From this juncture, saliva particles progress along an orthogonal unstable manifold characterized by a fractal-like pattern with backward folding along the upper limb. With each successive breathing cycle, newer saliva particles traverse the stable manifold until they converge at the saddle point of the vortex front, after which they navigate along the unstable manifold. Hence, these material lines, comprising both stable and unstable manifolds, emerge as significant factors in understanding particle dynamics.

## 6. Conclusion

This study presents a series of high-fidelity LES of human expiratory events, including coughing and normal breathing, to investigate the Eulerian and Lagrangian dynamics of breathing flows and the efficacy of face masks. Cough events were examined under both indoor and outdoor conditions, while normal breathing was studied exclusively indoors. Our simulations integrated a sharp-interface curvilinear immersed boundary method for human anatomy and a diffused interface immersed boundary method for face masks.

Our indoor cough simulations using the Eulerian method for the flow and saliva plume revealed that saliva particles exhaled during unmasked coughing remain suspended for extended periods and spread up to 2.62 m from the source. However, non-medical face masks substantially reduce this transport distance to 0.73 m. Evaporation was also shown to play a role in particle dispersion. Conversely, outdoor conditions facilitate the rapid transport of saliva particle plumes due to a mild breeze, rendering face masks ineffective. Our normal breathing simulation using the Eulerian method for both the flow and saliva plume showed that indoor unmasked breathing leads to the propagation of the saliva plume to distances up to 2.2 m in about 90 s time, surpassing CDC and WHO’s social distancing guidelines. Further, our findings showed that masked normal breathing would significantly curb the spreading process of the saliva particle plume, limiting the spreading distance to 0.72 m from the person. This marks the efficacy of face masks in suppressing the spreading of pathogen-carrying saliva particles under indoor conditions.

Our Lagrangian saliva particle tracking results for normal breathing revealed the formation of fractal-like Lagrangian coherent structures arising from particle trajectories. Based on their trajectories, saliva particles exhaled during indoor unmasked normal breathing traveled well beyond 2 m from the person. The Lagrangian saliva particle transport during masked breathing was, however, shown to be limited to short distances of 0.48 m from the person, corroborating our Eulerian simulation results. The numerically captured LCSs feature of the saliva particles vortex rings exhaled during unmasked normal breathing exhibit symmetric behavior for mouth-only breathing and asymmetry for breathing through the nose and mouth. This was shown to be due to transverse jet effects in the case of nose and mouth breathing. The use of LCS diagnostics and key material line identification, including Finite-Time Lyapunov Exponent (FTLE) fields, for mouth-only and nose-and-mouth breathing solidifies the significant role of material lines in saliva particle dynamics. Overall, our Eulerian and Lagrangian analyses underscore the extensive transport of saliva particles during expiratory events such as coughing and normal breathing, inviting continued research on expiatory saliva particle transport to enhance public safety and medical standards for future pandemics.
